# Evaluating the Feasibility of Performing Window of Opportunity Trials in Breast Cancer

**DOI:** 10.1155/2015/785793

**Published:** 2015-01-20

**Authors:** Angel Arnaout, Susan Robertson, Iryna Kuchuk, Demetrios Simos, Gregory R. Pond, Christina L. Addison, Mehrzad Namazi, Mark Clemons

**Affiliations:** ^1^Division of Surgical Oncology, Department of Surgery, Ottawa Hospital, Ottawa, ON, Canada; ^2^Cancer Therapeutics Program, Ottawa Hospital Research Institute, Ottawa, ON, Canada; ^3^Division of Anatomical Pathology, Ottawa Hospital, Ottawa, ON, Canada; ^4^Division of Medical Oncology, Department of Medicine, Ottawa Hospital and Ottawa Hospital Cancer Center, Ottawa, ON, Canada; ^5^Department of Oncology, McMaster University, Hamilton, ON, Canada

## Abstract

*Background*. The waiting period to surgery represents a valuable “window of opportunity” to evaluate novel therapeutic strategies. Interventional studies performed during this period require significant multidisciplinary collaboration to overcome logistical hurdles. We undertook a one-year prospective window of opportunity study to assess feasibility. *Methods*. Eligible newly diagnosed postmenopausal, estrogen receptor positive breast cancer patients awaiting primary surgery received anastrozole daily until surgery. Feasibility was assessed by (a) the proportion of patients who consented and (b) completed the study. Comparison of pre- and poststudy Ki67 labelling index and cleaved caspase 3 scores (CC3) was performed. *Results*. 22/131 (16.8%) patients were confirmed eligible and 20/22 (91%) patients completed the study. 19/20 (95%) patients agreed to undergo optional additional tissue biopsies. The mean duration of anastrozole use was 24.7 (15–44) days. There were a statistically significant decline in mean Ki67 indices of 48.8% (*p* < 0.001) and a trend towards significance in the decline of CC3 (*p* = 0.17) when comparing pre- with posttreatment values. *Conclusion*. window of opportunity trials in breast cancer are a feasible way of assessing the biologic efficacy of different therapies in the presurgical setting. The majority of eligible women were willing to participate including undergoing additional tissue biopsies.

## 1. Introduction

Window of opportunity (also called phase 0) trials can provide insight into biological effects and potential therapeutic efficacy of novel therapeutic strategies [1–4]. One example of window of opportunity trial is for women with newly diagnosed breast cancer to receive a study drug between the diagnostic breast biopsy and planned surgical resection. The advantage of window of opportunity trials is that they allow short-term testing of novel agents in patients who already have surgery planned as their primary therapy; and therefore agents may be tested in patients who are not pretreated. Window of opportunity trials differ from the more traditional neoadjuvant trials in that no therapeutic benefit is envisaged, whereas in neoadjuvant trials an investigational agent is given preoperatively along with chemotherapy or endocrine therapy for a longer duration (usually months) and surgery is delayed to allow for a therapeutic response in the tumor. Ultimately, window studies have the potential to expedite drug development process by improving the understanding of an agent's biologic effect early in its development through monitoring tissue samples obtained before and after drug exposure. These trials may assess target or pharmacodynamic effects of an intervention, allowing for greater potential to select for subsets of patients who might benefit from a therapy in clinical trials that are powered to detect changes in clinical outcome [2–4].

Despite the short duration of window studies, they are challenging to perform as they require close collaboration between multiple disciplines, including surgeons, oncologists, pathologists, radiologists, and laboratory scientists [2, 4–11]. In addition, one common concern of the preoperative window of opportunity model for patients and investigators is that it can lead to treatment delays if these evaluations cannot be completed within the standard normal surgical wait times [4–12]. As a result, window of opportunity studies are still relatively rare in the medical literature.

We undertook a one-year, pilot, window of opportunity trial using anastrozole to assess the feasibility of performing such trials at our institution. Feasibility was assessed through several endpoints, including the proportion of eligible patients, patient compliance, patient acceptability of additional research biopsies, and the ability to assess change in tumor Ki67 (marker of proliferation) and cleaved caspase 3 (CC3, marker of apoptosis).

## 2. Methods

### 2.1. Study Participants and Eligibility

This study was a single center, single arm, prospective study to assess the feasibility of performing a window of opportunity study at our center. The design was deliberately pragmatic and was designed to investigate the use of anastrozole in newly diagnosed postmenopausal, hormone receptor positive breast cancer patients awaiting primary surgery in the time from diagnostic tissue biopsy to surgery.

Eligibility criteria for the study included (1) postmenopausal status; (2) histologically confirmed estrogen receptor positive invasive carcinoma on diagnostic core biopsy; (3) the invasive cancer which was clinically and/or radiologically ≥2 cm in size; (4) patients who did not have any contraindications to take anastrozole; and (5) surgery date which was planned for 2–8 weeks after initial consultation. All patients had to be stage II or operable stage III as the practice in our institution is such that only inoperable stage III as well as stage IV patients went on to primary chemotherapy treatment. Patients could not have received hormone replacement therapy, tamoxifen, or an aromatase inhibitor within the previous 6 months or have known metastatic or recurrent breast cancer. Institutional Research Ethics Board and Health Canada approval was obtained prior to study commencement.

### 2.2. Study Procedures

**Figure 1:**
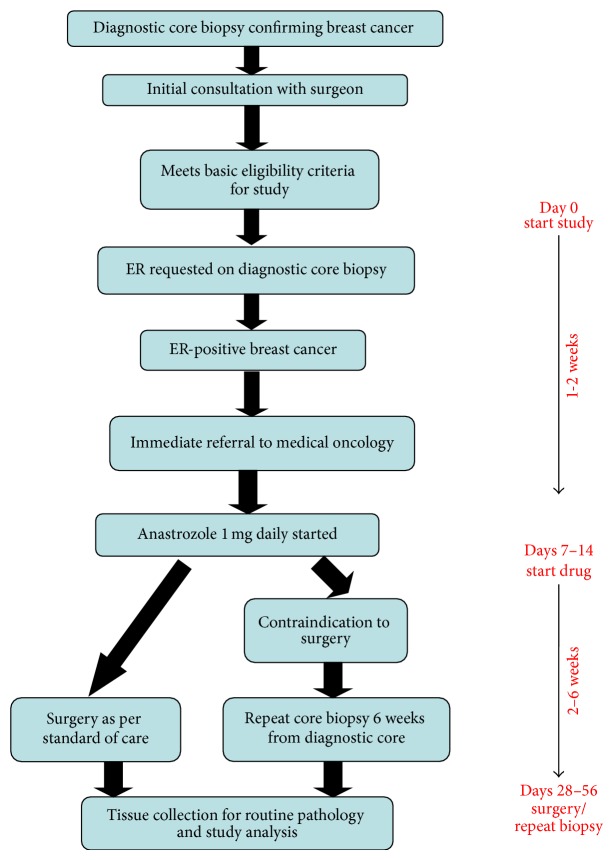
Study schema.

All potential study patients with a core biopsy confirmed invasive breast cancer were evaluated at initial consultation by one surgeon (AA). The surgeon decided whether the patient was potentially eligible based on tumor size and postmenopausal status. If the patient was interested in the study she was then approached for study screening by a research nurse for study eligibility (see Figure 1 study schema). Those patients who were screened and deemed potentially eligible had a formal request made to a pathologist (SR) for assessment of estrogen receptor (ER), progesterone receptor (PR), and human epidermal growth factor receptor 2 (HER2) on the diagnostic specimen. At the time of the study, routine biomarker analysis on initial diagnostic core biopsies was not performed at our institution and therefore could only be requested once the patient had consented to participate in the study. If ER and/or PR staining was greater than or equal to 1% they were considered positive and the patient was then eligible of the study.

All qualifying patients were referred to a medical oncologist (MC, IK, and DS) for assessment prior to starting anastrozole (1 mg po od). The time between starting anastrozole and surgery had to be a minimum of 2 weeks, and the last dose was to be taken the night before surgery. Patient compliance was assessed by pill count. Toxicity assessments (Common Terminology Criteria for Adverse Events (CTCAE) version 3.0 [7]) were performed prior to starting anastrozole, just before surgery, and 3-4 weeks after surgery.

### 2.3. Additional Optional Tissue, Blood, and Urine Collection

Patients with insufficient tissue in the initial diagnostic core biopsy for study analyses underwent an additional tumor biopsy. Even if there was sufficient initial core biopsy material for study analyses, at the time of the initial consent process, patients were also given the choice to undergo additional optional tissue biopsies and collection of blood and urine samples for use in the future as yet unplanned research. All tumor biopsies were immediately fixed in 10% neutral buffered formalin and excisional specimens were sliced and exposed to formalin within 1 hour with the majority having 24–72 hours of fixation time and less than 1/2 hour ischemic time. After standard tissue processing and embedding in paraffin wax sections were cut and were stained with hematoxylin and eosin or left unstained for immunohistochemistry.

### 2.4. Ki67 and CC3 Immunohistochemistry

Ki67 and CC3 were assessed on tissue sections cut from the FFPE diagnostic core biopsy (i.e., before anastrozole) and compared with expression in sections of surgical specimens as determined on selected representative tissue blocks (i.e., after anastrozole). The core biopsy specimens were generally 5-6 samples obtained with a 14 g needle. A minority of patients who were planned for surgery, and thus eligible for the study, were found to have medical comorbidities delaying their primary surgical treatment. For these patients, they continued on anastrozole while waiting for their surgery and a mandatory further core biopsy at 6 weeks was performed and used for the “postanastrozole” specimen.

Immunohistochemistry for Ki67 was performed using Leica PA0118 clone MM1 using the Refine Detection Kit from Leica. The “Ki67 index” (percentage of nuclei showing nuclear immunoreactivity of any intensity) was determined by computer image assisted count by a single pathologist (SR). In each case, after a low-power scan of the entire tissue section, hot spot regions of highest activity were selected and from these 1,000 tumor nuclei were counted at 400–600x magnification.

For CC3 immunohistochemical analysis, serial sections were reacted with cleaved caspase 3 (Asp175) specific antibody, New England Technology, using the Refine Detection Kit from Leica. Five hundred cells from each specimen under ×400 magnification in the best-stained tumor area of each section were counted by a single pathologist (SR) for each specimen. CC3 immunoreactivity score was defined as the percentage of stained cells.

### 2.5. Statistical Analyses

The following criteria were established by the trial investigators as being required in order to demonstrate a meaningful success of feasibility for the group: (1) accrual of >50% of patients who were approached and (2) successful completion of >50% of patients who initially received anastrozole. Descriptive statistics were used to summarize the Ki67 values at baseline, at time of surgery, and the relative change from baseline to surgery. The percentage Ki67 change is defined as [surgical Ki67 − baseline Ki67]/[baseline Ki67 ∗ 100%] for each patient and the absolute change is defined as surgical Ki67 − baseline Ki67. Hence, a negative value indicates a decrease in Ki67 from baseline to surgery. Similar analyses were performed for CC3. Spearman *ρ* was calculated to evaluate the association between caspase and Ki67, the change between these measures, and the association between the duration of drug and the change in these measures. All tests were two-sided, and *p* < 0.05 was considered statistically significant in all cases.

## 3. Results

### 3.1. Study Population and Characteristics

**Figure 2:**
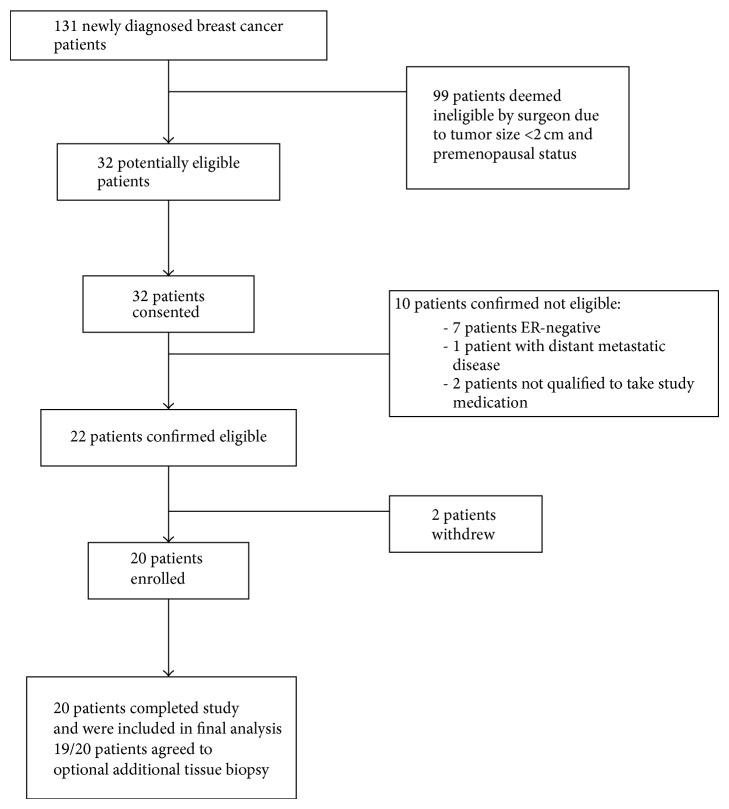
Patient flowchart.

Between September 2012 and September 2013, a total of 131 newly diagnosed breast cancer patients underwent initial surgical consultation (consort diagram, Figure 2). A total of 32 (24.4% of all patients) patients were deemed as being potentially eligible based on tumor size and menopausal status and thus were screened for the study. Of the 32 patients that were approached, all (100%) consented to participate in the study. There were 10 screen failures, seven were due to estrogen receptor negative status on the diagnostic core biopsy, one patient was found to have distant metastatic disease, and two patients did not qualify to take anastrozole due to medical comorbidities. 20/22 (91%) patients were therefore confirmed to be eligible for the study. Two patients withdrew from the study prior to taking anastrozole. Of the 20 remaining patients who received anastrozole, 100% completed the study.

Patient characteristics of the 20 patients that started anastrozole and completed the study are shown in Table 1. Mean patient age was 66.3 (range 52–89), and 80% of the patients had invasive ductal carcinoma. The mean tumor size was 3.8 cm (range 1.4–6.5 cm). The majority of patients had pathological stage II (T2N0) invasive ductal carcinoma and breast conserving surgery.

### 3.2. Duration of Treatment, Side Effects, and Compliance

The mean duration of drug intake was 24.7 days (SD 6.4 days; range 14–35 days), while the mean wait time from surgical decision to actual surgery date was 32.3 days (SD 8 days; range 15–44 days). The duration from the consent date to the patients' medical oncology appointment was a mean of 8.1 days (SD 4.6 days; range 1–19 days). All surgeries proceeded according to plan and scheduled date which was decided at the initial surgical consultation. Of the 20 patients that completed the study, 18/20 experienced mild to moderate adverse effects (grades 1-2) including hot flashes, joint pains, fatigue, and nausea. There were no grades 3 or 4 toxicities.

### 3.3. Changes in Tumor Ki67 and CC3

One patient had insufficient diagnostic tissue for baseline Ki67 and CC3 assessment and an additional tumor biopsy was performed prior to starting anastrozole. Another patient had sufficient core biopsy for pretreatment Ki67 but not CC3 analysis and also required an additional biopsy. The remaining 18 patients had sufficient paired pre- and posttreatment tissue samples for analysis. One patient did not proceed to surgery as planned within the 8-week time frame as she had ongoing cardiac comorbidities and more time was needed to better optimize her perioperative morbidity. She had a repeat biopsy 4 weeks after anastrozole treatment used for repeat Ki67 and CC3 analyses. After the additional biopsies, Ki67 and CC3 were assessable in all 20 patients from pre- and postanastrozole tumor tissue.

**Figure 3:**
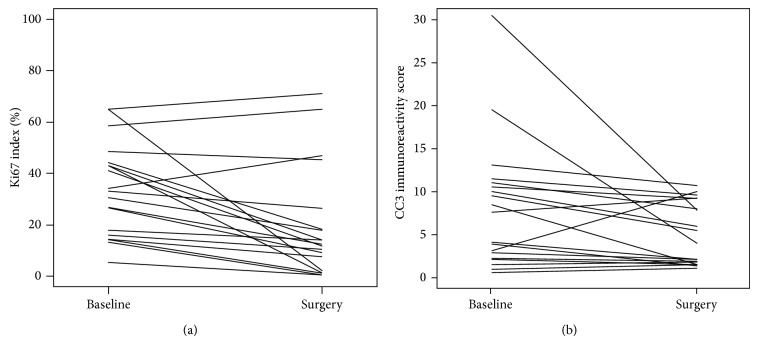
Ki67 labelling index (a) and cleaved caspase 3 (CC3) (b) at baseline and after anastrozole treatment at surgery.

Table 2 and Figure 3 summarize the Ki67 values before and after anastrozole treatment. One patient was excluded for analysis involving Ki67, as their postanastrozole Ki67 value increased by >1200%, which was an extreme outlier result and suggested technical inaccuracy; this patient was included for CC3 analyses.

Baseline pretreatment Ki67 mean was 33.2% (standard deviation 17.6%), compared to posttreatment Ki67 mean of 19.1% (standard deviation 21.2%) resulting in an absolute decline of 14.1% (*p* < 0.001) and relative decline of 48.8% (*p* = 0.001). 17/19 (89%) patients experienced a decline in the Ki67 value after treatment. Table 2 summarize the CC3 values before and after anastrozole treatment. Baseline pretreatment CC3 mean value was 7.7 (standard deviation 7.4) compared to posttreatment CC3 mean of 4.8 (standard deviation 3.6). This results in a statistically significant absolute decline of 2.9 points (*p* = 0.007) and a 10.5% relative decline that did not reach statistical significance (*p* = 0.17).  Table 3 shows the association between Ki67 and CC3 values at baseline and posttreatment. There was a weak-to-none association (*r* < |0.30| and *p* value > 0.05 for all) between Ki67 and CC3, at baseline, posttreatment, and for the change scores. Similarly, no association between Ki67 and drug duration was observed. However, for CC3, longer drug duration was moderately associated with a greater reduction in value, with a statistically significant association (*r* = −0.50, *p* value = 0.026) for relative percent change, and a trend towards significance for (*r* = −0.43, *p* value = 0.060) absolute change.

Results of any changes to Ki67 or CC3 as a result of the anastrozole treatment were blinded to the oncologist such that decisions regarding adjuvant chemotherapy or hormonal therapy decisions would not be affected.

### 3.4. Patient Acceptability for Additional Optional Tissue Collection

All 20 patients were approached to have additional optional breast tumor biopsies, additional blood sample retrieval, and urine collection for future research studies and 19/20 (95%) agreed to all three types of additional sample acquisition.

## 4. Discussion

While exciting for drug development strategies, performing window of opportunity trials faces multiple logistical and system barriers [4–13]. This albeit small pilot study demonstrated that performing such studies was possible at our cancer center. Our study did meet our established criteria for feasibility: we exceeded our target accrual of 50% of patients approached (32/32 patients approached consented to the study) and exceeded our 50% target completion rate in patients who received anastrozole (20/20 patients who received anastrozole complete the study). The results of our study demonstrated that women are willing to participate in such trials and undergo additional biopsies and give additional blood and urine samples for future research. Combined and closely coordinated efforts among the different disciplines involved in the patient's care (surgery, pathology, and radiology) meant that it was possible to conduct such trials without delaying surgery.

As feasibility to accrue patients involves a range of issues including eligibility criteria, patient compliance, and study mandated procedures we decided to use a number of feasibility measures. At our center ER, PR, and Her2 are not routinely performed on diagnostic core specimens. Therefore, in order to test for these potential patients had to sign consent* before *they were screened by a study research associate for eligibility. Clearly, depending on the eligibility criteria, the number of patients which must be approached to identify those likely meeting eligibility criteria for any given study will vary considerably. Even with our pragmatic design (newly diagnosed breast cancer, postmenopausal, >2 cm clinical or radiological confirmed, subsequently identified hormone receptor positive disease) 197 patients had to be approached or screened in order to identify the 32 potentially eligible patients who consented to the study. Subsequently, 69% (22/32) of the consented were eligible for the study, and 91% (20/22) ultimately completed the study. These numbers are similar to overall accrual rates in other window of opportunity studies [12–16]. We recognize that most of centers now routinely perform biomarkers on the diagnostic core biopsy specimens already, a process that was not in effect at our institution at the time of the study. The additional few days it took to obtain these results certainly may have helped allow for more time to enroll these patients without delay of their set surgical date, without which our accrual rate may have potentially been further reduced.

Our study also demonstrated that patient willingness to participate in such studies does not appear to be a barrier to accrual, as we were able to realize a high accrual rate. The fact that 19/20 (95%) patients enrolled agreed to undergo additional biopsies and blood and urine storage for future research studies reflected high patient enthusiasm for this type of research. This high rate of accrual likely reflects the fact that anastrozole, in addition to being a relatively safe and well tolerated drug, is already an established treatment for breast cancer, therefore making it a simple and easy drug to use for a pilot feasibility study [17, 18]. Its side effects did not preclude the patient from surgery which made it an acceptable agent for surgeons to consider. In the current study, the recognized therapeutic benefit of anastrozole likely helped patient compliance as all 20 patients who commenced anastrozole completed the study. Time will tell whether the use of agents that could interfere with surgical intervention (e.g., through effects on cardiac, neurological, marrow, coagulopathic, or thromboembolic events) would receive the same enthusiasm [1, 12]. Clearly, we do not know if a window of opportunity trial with an agent with no implied therapeutic advantage and unknown side effects will become more of an issue for patient compliance [4].

Changes in tumor Ki67 expression is a well-recognized surrogate endpoint for treatment response [18–21] and predictor of clinical outcome [22, 23]. Variability in Ki67 staining can occur as a result of a number of factors, including the duration of tissue ischemia, formalin quality, duration of fixation, immunohistochemical technique used, and assessor differences. Further, when comparing pretreatment biopsy to posttreatment excision or posttreatment biopsy, there may be effects of tumor heterogeneity on biomarker scores and at least a theoretical risk of alteration induced by the first biopsy procedure. We were able to have one pathologist (SR) perform all the analyses with the hope that variability in the assessor was reduced.

Many anticancer drugs induce apoptosis by molecular mechanisms mediated through mitochondrial dysfunction [24–26]. Release of cytochrome c from the internal part of the mitochondrial membrane into the cytosol results in the activation of caspase cascades, in particular caspase 9, caspase 3, caspase 6, and caspase 7. Because caspase 3 is the main executioner of apoptosis, immunohistochemical analysis to the active form of caspase 3, known as cleaved caspase 3 (CC3), has been used as an indicator of apoptosis in paraffin sections from various tissue sites [27–30]. Compared to the traditional TUNEL assay, whose interpretation and specificity have been reported as being difficult and controversial, CC3 immunohistochemistry is an easy, sensitive, and reliable method for detecting and quantifying apoptosis in tissues, with good correlation reported (*r* = 0.75) between it and the TUNEL assay [29]. Few studies have used it as a marker of response to treatment in breast cancer.

While the results may be counterintuitive in that CC3 (and therefore apoptosis) declined with anastrozole treatment, and a greater reduction was seen with longer duration of treatment, these results mirror what has been demonstrated with the TUNEL assay in anastrozole treated patients [20, 31, 32]. Unlike what is observed with cytotoxic chemotherapy, patients in the IMPACT study and others have demonstrated a decrease in apoptosis with endocrine therapy [20, 32]. It is possible that the capacity of breast cancer cells to pass into apoptosis is retarded by the profound antiproliferative effects of antiestrogenic therapy. It has been observed that* c-Myc* is a determinant of both proliferation and apoptosis [20], and its expression is enhanced by estrogen and suppressed by antiestrogens. This data suggests that estrogen may not be important for cell survival in breast cancers.

There remain a number of limitations to the current study. It was single center and single arm with a small sample size. Additional logistical and practical issues would be present in a multicenter or multiarm trial. Further difficulties may additionally be encountered if a novel agent with no known therapeutic benefit was used instead of anastrozole or if the biomarker was experimental and pathologists had little prior experience with measuring it. Additionally, the authors of this paper acknowledge that the involvement of a single dedicated surgeon and few medical oncologists would have potentially allowed for greater accrual. The success of the accrual may be less generalizable to larger group practices where it may be more difficult to overcome logistical hurdles. Finally, the authors recognize that the effect of presurgical hormonal therapy on Ki67 has already been demonstrated previously in studies such as IMPACT and POETIC [18, 20, 33]. The main objective of our study was to assess feasibility of such window trials at our institution.

In summary, this study demonstrates that accrual to nontherapeutic protocols is feasible in a single large academic cancer center and is acceptable to patients. The success achieved with this trial has been used as a strategy to convince other surgeons and patients to be involved in future research (NCT01948128).

## Figures and Tables

**Table 1:** Clinical and pathological characteristics of patients who enrolled in the study.

Patient and surgical specimen tumor characteristics	
Age (years), mean (SD)	66.3 (10)
Age range (years)	52–89
Invasive disease, *n* (%)	
Invasive, ductal	16/20 (80%)
Invasive, lobular	2/20 (10%)
Mixed invasive, ductal, and lobular	2/20 (10%)
Surgical tumor stage, *n* (%)	
T1	5/20 (25%)
T2	10/20 (50%)
T3	5/20 (25%)
Surgical nodal stage, *n* (%)	
N0	10/20 (50%)
N1	3/20 (15%)
N2	5/20 (25%)
N3	2/20 (10%)
ER, *n* (%)	
Positive	20/20 (100%)
Negative	0/20 (0%)
PR, *n* (%)	
Positive	17/20 (85%)
Negative	3/20 (15%)
HER2, *n* (%)	
Positive	4/20 (20%)
Negative	18/20 (80%)
Type of breast surgery	
Lumpectomy	11/20 (55%)
Mastectomy	9/20 (45%)

**Table 2:** Ki67 labeling index (%) and cleaved caspase 3 (CC3) at baseline (before anastrozole) and surgery (after anastrozole) on the 20 patients who completed the study.

	Statistic	Patients	*p* value
Ki67 labeling index (%)			
Baseline	Mean (std)	33.2 (17.6)	
	Median (10p, 90p)	31.7 (13.2, 61.8)	
End of study	Mean (std)	19.1 (21.2)	
	Median (10p, 90p)	12.1 (0.7, 55.8)	
% change	Mean (std)	−48.8 (40.1)	
	Median (10p, 90p)	−55.8 (−97.5, 10.1)	*p* = 0.001
Absolute change	Mean (std)	−14.1 (17.5)	
	Median (10p, 90p)	−12.2 (−35.6, 6.2)	*p* < 0.001
Cleaved caspase 3 (CC3)			
Baseline	Mean (std)	7.7 (7.4)	
	Median (10p, 90p)	5.9 (1.3, 16.3)	
End of study	Mean (std)	4.8 (3.6)	
	Median (10p, 90p)	3.1 (1.2, 9.8)	
% change	Mean (std)	−10.5 (67.7)	
	Median (10p, 90p)	−25.5 (−76.9, 66.7)	*p* = 0.17
Absolute change	Mean (std)	−2.9 (6.3)	
	Median (10p, 90p)	−1.6 (−11.2, 1.1)	*p* = 0.007

10p = 10th percentile; 90p = 90th percentile; *p* value = Wilcoxon rank sum; std = standard deviation.

**Table 3:** Association between Ki67 labelling index and cleaved caspase 3 (CC3) values at baseline (before anastrozole) and surgery (after anastrozole) using the Spearman *r* (*p* value) for associations.

	Baseline Ki67	Surgical Ki67	% change Ki67	Absolute change Ki67	Drug duration
Baseline CC3	−0.25				
	(0.14)				

Surgical CC3		0.06			
		(0.74)			

% change CC3			−0.02		−0.50
			(0.90)		(0.026)

Absolute change CC3				−0.17	−0.43
				(0.33)	(0.060)

Drug duration			0.23	0.07	
			(0.32)	(0.76)	
